# TIA: transient brain ischemia but persistent damage

**DOI:** 10.1038/s44321-026-00404-w

**Published:** 2026-03-20

**Authors:** Pei Zheng, Fu-Dong Shi, Kaibin Shi

**Affiliations:** 1 Department of Neurology, Beijing Tiantan Hospital, Capital Medical University, Beijing, China; 2 Department of Neurology, Tianjin Medical University General Hospital, Tianjin, China; 3 Chinese Institute for Immunology, Chinese Institutes for Medical Research, Beijing, China

**Keywords:** Neuroscience

## Abstract

This News & Views highlights a recent study by Llovera and colleagues (in this issue of *EMBO Molecular Medicine*) introduces a rigorously characterized, tissue-based animal model of transient brain ischemia.

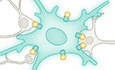

## Teaser

Transient ischemia attack (TIA) is a brief episode of neurological dysfunction caused by a transient but reversible disruption of blood supply to a specific region of brain without demonstrated tissue injury. Although TIA has long been regarded as a benign cerebral ischemia event, it in fact represents a critical warning for patients and their caregivers, as approximately 23% of patients with ischemic stroke experience TIA hours to days before the onset of acute cerebral infarction (Rothwell and Warlow, 2005). In addition, TIA is associated with long-term cognitive decline, which is a higher risk of dementia (Del Bene et al, 2025). Therefore, timely recognition and management of TIA are essential and more valuable to prevent subsequent stroke and reduce the risk of disability.

TIA remains underexplored, in part due to the absence of overt tissue injury and the lack of well-defined experimental models for TIA research. Llovera and colleagues address this gap by establishing a rigorously characterized, tissue-based TIA model using a brief five-minute middle cerebral artery occlusion (Llovera et al, 2026). In this model, transient behavioral and cognitive deficits emerge in the absence of detectable neuronal loss, blood–brain barrier disruption, or metabolic impairment, closely mirroring the clinical definition of TIA. Importantly, this model reframes TIA as a disorder of functional network integrity rather than structural brain damage, revealing persistent, region-specific cortical connectivity deficits, particularly within middle cerebral artery–supplied somatosensory circuits, accompanied by synaptic alterations despite preserved neuronal survival.

**Figure 1:**
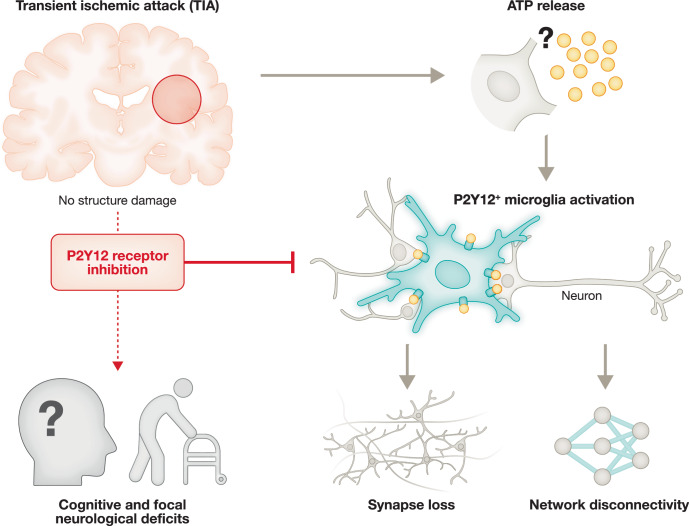
Microglial activation and neuronal dysfunction in TIA. Transient disruption of blood flow to the brain leads to the release of ATP to the extracellular space, which activates microglia via P2Y12 receptors, leading to a morphological change of microglial cells and subsequent enhanced microglial-neuron interactions. This enhanced microglial-neuron interaction resulting synapse loss and network dysconnectivity, causing neuronal dysfunction post-TIA, potentially causing cognitive impairment after TIA. Blocking ATP-induced microglial activation via a P2Y12 inhibitor preserved neuronal function and improved the neurological outcome of TIA.

Using this tissue-injury–free TIA model, the authors further investigated the mechanisms linking transient ischemia to persistent functional alterations. This revealed stress- and inflammation-related alterations in the extracellular milieu following TIA, accompanied by a transcriptional response dominated by microglia that evolves toward an activated and dynamically engaged state. Transient elevations in extracellular ATP were followed by rapid recruitment of microglial processes through the purinergic receptor P2Y12, and pharmacological inhibition of P2Y12 normalized aberrant microglia–synapse interactions while preventing both focal neurological and cognitive deficits (Fig. 1). Together, these findings delineate an ATP–P2Y12–microglia signaling cascade through which transient ischemia leaves a lasting immunologically mediated imprint on neural circuits, leading to synaptic disruption and network dysfunction in the absence of overt tissue injury, an effect that is further exacerbated with aging.

Although this study provides compelling evidence that ATP-driven microglial reactivity can acutely disrupt synaptic integrity and cognitive performance after TIA, the implications for long-term cognitive risk remain unresolved. Notably, cognitive deficits were assessed only within two days after model induction, leaving open whether these impairments are fully reversible or represent an early step toward progressive cognitive decline and dementia. Clinical studies have reported persistent cognitive impairment months to years after TIA, often accompanied by alterations in white matter microstructure and thalamocortical connectivity despite the absence of infarction (Tariq et al, 2020). These observations suggest that distributed network dysfunction, involving white matter tracts and subcortical hubs may play a central role in post-TIA cognition. How acute cortical microglial responses interface with longer-lasting circuit- and connectivity-level alterations therefore remains an important unanswered question.

Overall, the experimental evidence provided by this study demonstrates that TIA can induce persistent neuronal functional alterations in the absence of overt tissue injury. To validate these findings in a clinical setting, image microglia with TSPO tracer in TIA patients, and compare findings generated in this model, especially in a longitudinal fashion, is an appealing study. Furthermore, for therapeutic purpose, preclinical and clinical modulation of microglial function will be interesting and worth to try, in addition to blocking P2Y12 receptor as suggested by the present study, Brunton kinase inhibitor (Fox et al, 2025), anti-TREM2 treatment, FPR1 antagonist (Li et al, 2025), as well as CSFR1 inhibitors, as raised by recent progresses both in clinical and preclinical evidence, are potential valuable candidates.

Given the notoriously low success rate of translating therapeutic strategies from experimental stroke models to clinical practice, this rigorously characterized TIA model offers an important opportunity to re-examine early, subclinical mechanisms relevant to ischemic brain dysfunction. At the same time, the heterogeneity of human TIA poses a major challenge for translation. The emerging in silico and digital twin approaches may offer a complementary avenue. Mechanism-informed computational models, increasingly coupled with machine learning, enable the integration of vascular dynamics, cellular responses, and network-level brain function to simulate how brief ischemic perturbations propagate across biological scales. In stroke research (Li et al, 2024; Monclova et al, 2025), digital twin frameworks have begun to capture patient-specific anatomy, perfusion, and treatment responses, highlighting the feasibility of modeling biological variability beyond what is achievable in conventional experimental systems. Applied to TIA, such approaches could be particularly valuable for interrogating how transient ischemia engages immune-responsive cell types, reshapes synaptic and network dynamics, and interacts with age and comorbidities to influence divergent cognitive outcomes. Although still at an early stage, such models will need to be further refined and constrained by biological data.
